# A Dose-Response Relationship to Radiotherapy for Cutaneous Lesions of Langerhans Cell Histiocytosis

**DOI:** 10.1155/2021/6680635

**Published:** 2021-03-24

**Authors:** Mark K. Farrugia, Carl Morrison, Francisco Hernandez-Ilizaliturri, Saif Aljabab

**Affiliations:** ^1^Department of Radiation Medicine, Roswell Park Comprehensive Cancer Center, USA; ^2^Department of Pathology, Roswell Park Comprehensive Cancer Center, USA; ^3^Department of Medicine-Lymphoma/Myeloma, Roswell Park Comprehensive Cancer Center, USA

## Abstract

Langerhans cell histiocytosis (LCH) is a rare disease, afflicting approximately 4.6 and 1-2 per 1 million children and adults, respectively. While LCH can involve numerous organ systems such as the lung or bone, it is uncommon for the disease to be limited to the skin. Radiotherapy has an established role for osseous lesions. However, the efficacy and dose for nonosseous manifestations of the disease are not well described. In the current case report, we detail a 49-year-old adult male with skin-limited LCH requiring palliative radiotherapy (RT) to numerous sites for pain control. The patient was initially diagnosed and treated with single agent cytarabine for approximately 6 months. Despite treatment, he had little symptomatic response of his cutaneous lesions. We delivered a single dose of 8 Gray (Gy) to 3 separate skin lesions, including the bilateral groin, right popliteal region, and right axillary lesion, which resulted in pain reduction and partial response at four-month follow-up. Subsequently, we decided to treat the left axillary untreated lesion to a higher dose of 24 Gy in 12 fractions. At four-month follow-up, the left axilla RT resulted in complete clinical response and improved pain control compared to the right axilla. Following RT treatments, the patient was found to have a BRAF mutation, and vemurafenib was initiated. Further follow-up with positron emissions tomography demonstrated complete metabolic response in numerous disease areas, including both axillae. Based on this case report's findings, a higher radiotherapy dose may be more effective for treating cutaneous LCH.

## 1. Introduction

Langerhans cell histiocytosis (LCH) is a rare disease, afflicting approximately 4.6 and 1-2 per 1 million children and adults, respectively [1, 2]. LCH causes a granulomatous inflammatory infiltrate which can damage any organ system; however, the bone, skin, lungs, and pituitary are particularly susceptible. In an international registry of adult LCH patients, 31.4% presented with single system disease where the most common individually involved sites were the lung (51.1%), bone (38.3%), and skin (7%) [3]. Initial treatment typically includes systemic therapy, and initial regimens can vary [4, 5] For patients who do not respond to treatment, the clinical manifestations of LCH, especially in the skin and bones, can be quite morbid. In these instances, radiotherapy (RT) can be useful for palliation of these symptoms. However, given the rarity of this illness, there is limited data regarding the radiotherapeutic regimens to employ for these patients, which often describe a wide range of radiation doses avaiable [6–9]. In this case report, we describe an adult patient with a single system, multisite refractory cutaneous LCH who underwent multiple courses of RT for different skin lesions, demonstrating a dose relationship between RT regimen and clinical response.

## 2. Case Report

The patient was a 49-year-old male with a history of chronic hidradenitis involving the axillae and inguinal folds. He had a past medical history of hypertension, type II diabetes mellitus, morbid obesity status post sleeve gastrectomy, and chromophobe pituitary adenoma status post transsphenoidal resection. The patient was a never smoker and denied any marijuana use. Prior to presentation, patient experienced ulcerative skin lesions within the axillae and inguinal folds causing a nagging, throbbing pain with associated intermittent drainage over a period of 5 years. He was previously treated with adalimumab for a year and a half, as well as doxycycline and prednisone 5 mg daily with no improvement in his chronic hidradenitis. Given the recalcitrant nature of his chronic hidradenitis, he underwent a right axilla vault skin biopsy consistent with a diagnosis of LCH. He was subsequently referred to medical oncology where he underwent additional workup.

Panel for pituitary function revealed lower than normal testosterone (89 ng/dL) and free testosterone (2.7 ng/dL); however, follicle stimulating hormone, luteinizing hormone, thyroid stimulating hormone, thyroxine, cortisol, and adrenocorticotropic hormone levels were within normal limits. Additional blood work demonstrated a mild anemia (hemoglobin 12.3 g/dL) without significant derangements in white blood cell counts and lactate dehydrogenase levels. Bone marrow biopsy was negative for disease, magnetic resonance imaging of the brain did not show any evidence of LCH associated lesions, and diagnostic computed tomography (CT) imaging of the chest did not demonstrate any pulmonary involvement of LCH. Skull base to mid-thigh positron emission tomography with diagnostic computed tomography (PET/CT) revealed hypermetabolic adenopathy in the bilateral iliofemoral, prepectoral, and right axillary regions with avidity observed in the skin folds of the arms and chest wall. Based on these findings, the patient was diagnosed with skin-limited LCH.

**Figure 1:**
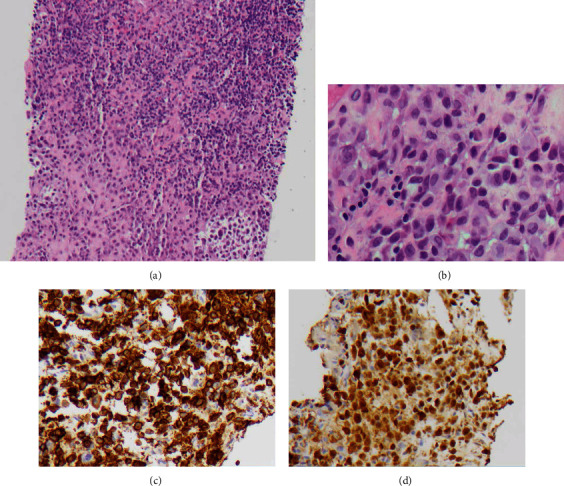
Hematoxylin and eosin (H&E) and immunohistochemical (IHC) staining. (a) H&E of needle core biopsy at low power showing aggregates of Langerhans cells surrounded by small mature lymphocytes. (b) H&E of LCH cells with oval nuclei showing features of nuclear grooves, occasional binucleation, and abundant cytoplasm without granules. (c) IHC for CD1a showing strong diffuse cytoplasmic staining with membranous accentuation. (d) IHC for S-100 showing variable strong to weak cytoplasmic staining.

**Figure 2:**
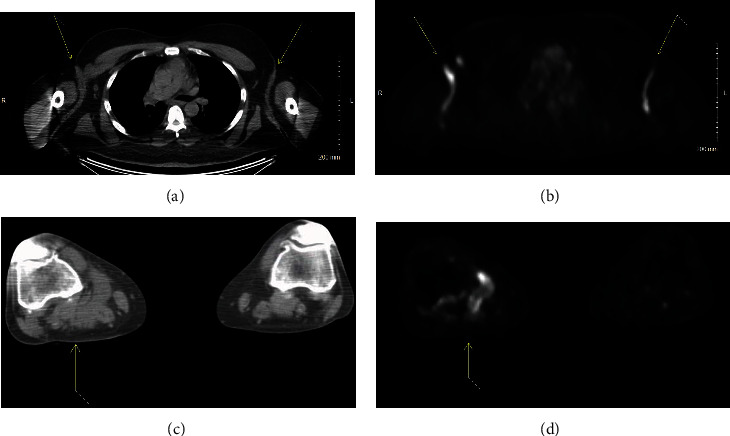
PET/CT imaging prior to starting radiotherapy. Axial CT of patient axilla (arrows) (a) and corresponding PET image (b). Axial CT of patient's right popliteal mass (arrow) (c) and corresponding PET image (d).

Three months after diagnosis, the patient was started on single agent, low-dose cytarabine 100 mg/m^2^ days 1-5 every 28 days for 6 cycles. At the start of his second cycle of cytarabine, the patient complained of right leg swelling and was found to have a right popliteal mass. US-guided biopsy demonstrated LCH cells with oval nuclei showing features of nuclear grooves, occasional binucleation, and abundant cytoplasm without granules as well as strong diffuse cytoplasmic staining for CD1a and variable weak to strong cytoplasmic staining for S-100 (Figure 1). Prechemotherapy imaging did not evaluate this region; therefore, it was unclear if it developed on treatment. The decision to continue therapy was made, and end of therapy PET/CT imaging demonstrated a mixed metabolic response to treatment with persistent avidity demonstrated in the previously described regions, including the right popliteal (Figure 2). Clinically, the patient was still experiencing irritation of bilateral axilla, groin/pannus, and right knee. The patient was subsequently referred to radiation oncology to discuss treatment options.

**Figure 3:**
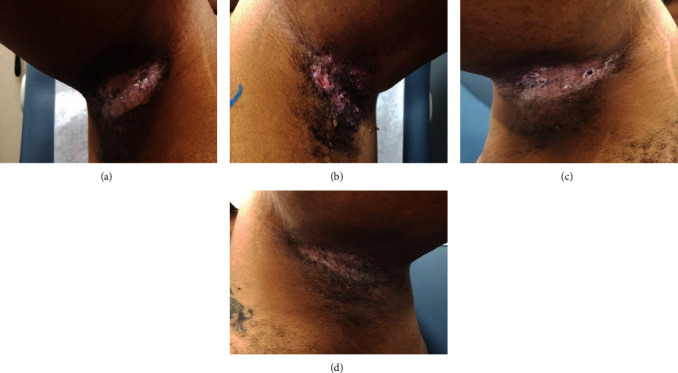
(a) Right axillary lesions 3 weeks after RT (8 Gy in 1 fraction). (b) Left axillary lesions prior to RT. Clinical response following RT for the right axilla (5-month posttreatment) (c) and left axilla (4-month posttreatment) (d).

**Figure 4:**
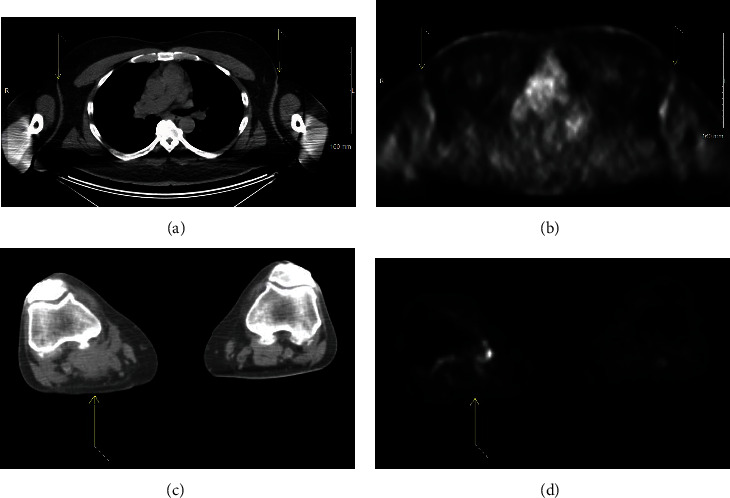
PET/CT imaging approximately 7 months after starting RT. Axial CT of patient axilla (arrows) (a) and corresponding PET image (b). Axial CT of patient's right popliteal mass (arrow) (c) and corresponding PET image (d).

During consultation, he endorsed continued irritation of the aforementioned skin folds, with the right popliteal, groin/pannus, and right axilla as the most symptomatic. On exam, he had ulcerative, violaceous lesion in the groin, pannus, and axilla. No mass could be palpated in the right popliteal region. Due to multiple areas of involvement, we offered palliative RT, 8 Gy in 1 fraction to the most symptomatic lesions at that time which included the right popliteal mass, the groin/pannus skin lesions, and right axilla skin lesion. Each of the three sites was treated in a single session using 3-dimensional conformal RT (3DCRT). Approximately 3 weeks following delivery of his RT, the patient was seen in follow-up. At that time, he described an improvement in pain and discharge of the treated skin lesions (Figure 3(a)). On exam, the right axilla had decreased in size with less ulceration, no mass was palpable in the right popliteal region, and he had stable lesions in the left pannus. However, his left axilla was becoming more bothersome (Figure 3(b)) and it showed similar ulcerative lesions with associated discharge. Since the right axilla skin ulceration did not completely resolve with 8 Gy, we decided to give a higher dose of 24 Gy in 12 fractions using 3DCRT. During this period, molecular testing was performed which detected the presence of BRAF mutation and approximately 2 months post completion of his second course of RT; he was started on vemurafenib. The patient was seen in follow-up 4 months after completing his last fraction of RT with no evidence of disease in his left axilla (Figure 3(d)). By comparison, the right axilla had stable residual disease (Figure 3(c)). The patient continued to follow with medical oncology, and PET/CT obtained (7 months after RT, 5 months duration of vemurafenib) revealed complete metabolic response of the axilla (Figures 4(a) and 4(b)) and stable mixed response in the popliteal region (Figures 4(c) and 4(d)). To date, the patient continues to receive vemurafenib.

## 3. Discussion

LCH is a rare disease, with the majority of patients presenting as children [10]. The role of radiotherapy in LCH has been described as early as 1979, where Greenberger et al. detailed the management of LCH-involved bone utilizing radiation doses ranging between 1 and 20 Gy with excellent local control [11]. Contemporary approaches using RT in LCH over the past 20 years are summarized in Table 1.

The clinical presentation of LCH can be heterogeneous with some patients presenting with a single site of disease, while others can have multisite of a single organ system or multisystem involvement. In the current case report, the patient presented with multisite, single organ disease. While he had a history of pituitary dysfunction, this appeared unrelated to his subsequent diagnosis of LCH. Skin-only LCH is less common than single-system bone disease but however has been described to occur in approximately 13% of patients [12].

While Arico et al. reported the lung to be the commonly involved site in single-system disease, they only report management with systemic treatment and do not describe the percentage of patients who received RT. Other series focusing on patients managed with RT tend to have higher proportions of osseous disease [3]. Both the extent and site of disease have prognostic implications with regard to response to RT and event-free survival. For example, Kriz et al. found 37.7% of patients to have a single site of bone involvement, 46.4% had multiple bony sites, and 15.9% of patients had organ involvement [7]. The radiographic control rate of all lesions using RT was 91.4%. When compared to a single site of disease, those with multiple bone lesions had worse overall survival while patients with organ involvement did substantially worse than both groups. Furthermore, in an institutional review by Laird et al., it was found that 79% of patients have single-system disease with the bone as the most common site involved [8]. In this study, complete symptomatic response was observed in 89% of patients with bone lesions at 1 year compared to 50% for nonosseous lesions following RT. [8] Moreover, nonbone lesions were found to have a 3-year freedom from local failure of 63.2% where no events were observed in bony lesions. In addition, those with nonbone lesions had significantly worse distant progression-free and overall survival. Similarly, Olschewski and Seegenschmiedt found those with bony lesions alone to achieve complete remission in 93% of cases with RT compared to 76% complete remission in patients with multisystem disease [9].

A wide spectrum of doses (2.0-50.4 Gy) has been used to treat LCH-involved sites; however, the most common median doses range between 10 and 24 Gy (Table 1). In the current patient, an initial dose of 8 Gy in a single fraction was chosen, which is a frequently used regimen for palliation and an accepted dose for palliative RT for hematologic malignancies such as myeloma [13]. In contrast, 24 Gy in 12 fractions was used for the second course of treatment, which is commonly used in non-Hodgkin's lymphoma and myeloid-derived cancers [14, 15]. In the current patient, 24 Gy was associated with a superior clinical and symptomatic response as compared to 8 Gy to the contralateral axilla. While a previous study has shown excellent outcomes with low-dose RT for LCH, others favor median doses > 12 Gy [6–9, 16, 17]. Given that these were soft tissue lesions which are inherently less sensitive to RT, it is possible the additional dose and/or fractionation was particularly helpful in this setting.

In addition to the comparative effects of RT dose, the patient demonstrated a compelling and durable response to the small molecule inhibitor vemurafenib. Previously, BRAF mutations were found to be present in 57% percent of achieved specimens, and as such, BRAF inhibition has been explored as a potential therapeutic option in LCH [18] In a phase 1/2 trial of LCH and Erdheim-Chester disease patients, vemurafenib elicited a 41% response by Response Evaluation Criteria in Solid Tumors (RECIST) [19]. Furthermore, a number of case reports have demonstrated efficacy of BRAF inhibition in pediatric LCH [4]. Despite these findings and the high incidence of BRAF mutations in LCH, BRAF inhibition remains a subject of investigation in these patients.

While these findings are restricted to a single patient with short interval follow-up, the following case report suggests a dose response relationship regarding RT for the LCH-involved skin in an adult patient, supporting the use of higher doses such as 24 Gy in this relatively resistant subsite of disease.

## Figures and Tables

**Table 1:** Prior reports on the use of radiotherapy in Langerhans cell histiocytosis.

Author	Year	Type	*n*	Median dose (Gy)	Dose range (Gy)	LC (%)
Hristov et al.	2011	Case report	1	24	—	100
Kriz et al.	2013	Retrospective	80	15	3.0-50.4	77
Laird et al.	2018	Retrospective	39	11.4	7.5-50.4	87
Olschewski et al.	2006	Retrospective	98	24	2.0-40	77.5
Meyer et al.	2012	Case report	1	9	—	100
Kotecha et al.	2014	Retrospective	69	10	2.5-45	91.4

## Data Availability

The data used to support the findings of this study are included within the article.
